# Blocked Presentation Leads Participants to Overutilize Domain Familiarity as a Cue for Judgments of Learning (JOLs)

**DOI:** 10.3390/jintelligence11070142

**Published:** 2023-07-17

**Authors:** Michael J. Serra, Lindzi L. Shanks

**Affiliations:** Department of Psychological Sciences, Texas Tech University, Lubbock, TX 79409, USA; lindzi.shanks@gmail.com

**Keywords:** metacognition, domain familiarity, judgments of learning (JOLs), accuracy, overconfidence, cue utilization

## Abstract

The accuracy of judgments of learning (JOLs) is vital for efficient self-regulated learning. We examined a situation in which participants overutilize their prior knowledge of a topic (“domain familiarity”) as a basis for JOLs, resulting in substantial overconfidence in topics they know the most about. College students rank ordered their knowledge across ten different domains and studied, judged, and then completed a test on facts from those domains. Recall and JOLs were linearly related to self-rated knowledge, as was overconfidence: participants were most overconfident for topics they knew more about, indicating the overutilization of domain familiarity as a cue for JOLs. We examined aspects of the task that might contribute to this pattern, including the order of the task phases and whether participants studied the facts blocked by topic. Although participants used domain familiarity as a cue for JOLs regardless of task design, we found that studying facts from multiple topics blocked by topic led them to overutilize this cue. In contrast, whether participants completed the rank ordering before studying the facts or received a warning about this tendency did not alter the pattern. The relative accuracy of participants’ JOLs, however, was not related to domain familiarity under any conditions.

## 1. Introduction

Learners are not blank slates, and they are not all equally able to learn new content (cf. Metcalfe 2002). One factor that can affect students’ ability to learn new information is domain familiarity (also known as domain knowledge): the level of knowledge about a topic or domain that learners already have when they encounter new information to learn from that same topic (cf. Dunlosky et al. 2013). In the present studies, we examined the accuracy of people’s metacognitive judgments as they learned facts in domains they knew more and less about.

As Witherby and Carpenter (2022) described, the relationship between how much one already knows about a topic and how easily one can learn new information about that topic often reflects a “rich get richer” outcome: people who already know more about a topic can learn more about it compared to people who know less (cf. Boscolo and Mason 2003; Carpenter et al. 2018; Ericsson and Kintsch 1995; Feltovich et al. 2006; Griffin et al. 2009; McNamara et al. 1996; Miller et al. 2004; Shanks and Serra 2014; Wiley 2005). Researchers have considered the positive relationship of domain knowledge to learning and whether it is related to people’s ability to judge their learning across or within domains. Although studies have been inconsistent as to how domain knowledge relates to metacognitive monitoring (e.g., Glenberg and Epstein 1987; Glenberg et al. 1987; Griffin et al. 2009; Maki 1998; Sanocki 1987; Shanks and Serra 2014; Toth et al. 2011), in at least some cases, domain familiarity is associated with significant errors in people’s monitoring of their learning. These errors include an “unskilled and unaware” pattern in which learners greatly overpredict their test performance for content they do not know well (cf. Kruger and Dunning 1999; Serra and DeMarree 2016) and an “overutilization of domain familiarity” pattern in which they greatly overpredict their test performance for content they do know well (cf. Shanks and Serra 2014). In the present studies, we replicated the overutilization of domain familiarity pattern with people judging their learning for facts across 10 different domains and examined some potential reasons for why the pattern occurs. The results not only have implications for understanding why this specific inaccuracy occurs, but also for understanding how people make metacognitive judgments in general.

### 1.1. The Monitoring of Learning

The term metacognition refers to people’s knowledge, monitoring, and control of other mental states or cognitive processes (cf. Dunlosky and Metcalfe 2008). For example, students studying for an exam might monitor their current learning and conclude that they are not yet ready to take an upcoming exam, have knowledge about effective study strategies they could use to study, and engage in control when they strategically allocate the remaining time in the week before the exam to devote more time for studying topics that they do not understand well. Of course, any error in knowledge, monitoring, or control, while studying could have negative downstream effects on the efficacy of the student’s endeavor (cf. Serra and Metcalfe 2009); they might choose an ineffective study strategy or make unproductive restudy decisions (cf. Finn 2008; Metcalfe and Finn 2008; Shanks and Serra 2014), or they might not reserve enough time to study all the content they needed to review before the exam (cf. Bjork et al. 2013; Dunlosky and Rawson 2012; Grimes 2002; Kornell and Bjork 2008b; Kornell and Metcalfe 2006). Additionally, inaccurate metacognitive monitoring can have negative consequences beyond studying for a single exam or for success in a single course. Greatly overestimating one’s learning or abilities in a domain leads students to doubt their choice of major, to change majors, and even to drop out of college entirely (cf. Grimes 2002; Stinebrickner and Stinebrickner 2014).

#### 1.1.1. Measures of Metacognitive Accuracy

People make metacognitive judgments inferentially, using “cues” about the cognitive process under consideration to make their judgments (cf. Dunlosky and Tauber 2014; Koriat 1997). To the extent that those cues are predictive of subsequent performance and that people have factored them into their metacognitive judgments appropriately, their judgments should be correspondingly predictive of performance, or “accurate” (cf. Serra and Metcalfe 2009).

In the present studies, we examined an error in a particular type of metacognitive judgment: judgments of learning (JOLs). These are prospective judgments about memory or learning that a person makes in between the encoding and testing of target information (Dunlosky et al. 2007). For example, after studying the history fact, “Sandra Day O’Connor was the first woman member of the U.S. Supreme Court”, researchers might ask participants to make a JOL indicating the likelihood that they will be able to answer a question about that information on a subsequent test. Most often, this is performed using a continuous probability scale, such as having participants indicate the likelihood (on a 0% to 100% scale) that they feel they would be able to answer such a question later.

Two common measures of JOL accuracy (cf. Dunlosky et al. 2007; Serra and Metcalfe 2009) are directly relevant to the present studies. The first, absolute accuracy, refers to a difference score calculated by subtracting participants’ mean recall from their mean JOL. When the mean JOL is significantly higher than recall, we would say the judgments are “overconfident”, and when the mean JOL is significantly lower than recall, we would say the judgments are “underconfident”. When the mean JOLs and recall do not differ, we would say that the judgments are “accurate” or “calibrated”. Here, we focused on a particular illusion of metacognitive monitoring that can cause learners to greatly overestimate how well they know the information they are trying to learn.

The second, relative accuracy, refers to participants’ ability to discriminate learned from unlearned items with their JOLs. A common way to assess relative accuracy involves calculating a gamma correlation across items between a participants’ JOLs and recall outcomes (Dunlosky et al. 2007). The value of this measure can range from +1.0 to −1.0. To the extent that participants show good discrimination between learned and unlearned items (i.e., assigning higher JOLs to items they will recall and lower JOLs to items they will not recall), relative accuracy will be positive and of higher magnitude. If participants show little or no discrimination between learned and unlearned items, it will be closer to zero (or might even be incalculable). Finally, negative values would indicate extremely poor accuracy in the form of inverse discrimination (i.e., assigning lower JOLs to items they will recall and higher JOLs to items they will not recall). In the present studies, we sought to provide additional data to the question of whether (and how) relative accuracy relates to domain knowledge.

#### 1.1.2. The Overutilization of Domain Familiarity as a Cue for JOLs

Given that people’s learning of new information across domains is often positively related to how much information they already know from those domains, it makes sense for them to use domain knowledge as a cue for JOLs. People often—appropriately—give higher JOLs to new information they are learning from topics they know more about than topics they know less about (cf. Glenberg and Epstein 1987; Griffin et al. 2009; Shanks and Serra 2014; Toth et al. 2011; Witherby and Carpenter 2022; Witherby et al. 2023). Often, such findings reflect JOLs that participants make for only two topics, so the question is primarily whether they gave higher JOLs to one topic than the other (rather than absolute accuracy per se).

**Figure 1:**
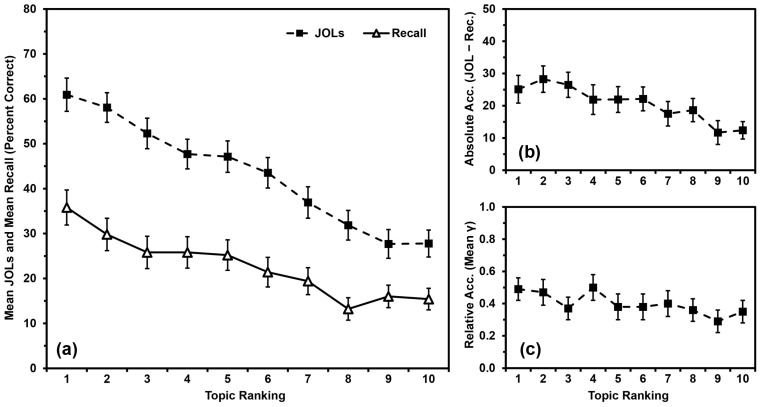
Data from Shanks and Serra (2014), Experiment 1: (**a**) Mean JOLs and mean recall performance (percent correct) by topic ranking; (**b**) Mean absolute accuracy (JOLs minus recall) by topic ranking; (**c**) Mean relative accuracy (gamma correlations) by topic ranking after inputting 0 s for missing values. Error bars are one standard error of the mean.

In contrast, Shanks and Serra (2014) had participants rank order their knowledge across 10 different domains (atmospheric science, biology, business, chemistry, English, history, math, performing arts, psychology, and sociology) from best known to worst known. They then studied and made JOLs for 10 facts from each topic. To date, this might be the only set of studies to examine domain familiarity for so many topics at the same time (i.e., information from 10 different topics rather than one or two topics). Participants’ recall of facts from the topics was linearly related to their self-rated preexisting knowledge in those topics, as were their JOLs (Figure 1a). Participants generally overestimated their recall of the facts, but overconfidence was most pronounced for topics that participants felt they knew the most about (Figure 1b). More specifically, the slope of participants’ JOLs was of greater magnitude than was the slope of their recall compared to domain familiarity (Figure 1a; see also Glenberg et al. 1987 for a similar outcome). Shanks and Serra (2014) dubbed this pattern “the overutilization of domain familiarity as a cue for JOLs”. Of course, it is both logical and appropriate for participants’ JOLs to be linearly related to their domain familiarity, even if their judgments tend to show pervasive overconfidence in this paradigm. The aspect of the judgment (in)accuracy that we are concerned with in the present studies is that participants are most overconfident in the topics they know the best. For simplicity, we will refer to this outcome as “the overutilization pattern”.

As noted earlier, participants make JOLs inferentially, but we do not know exactly how they utilize their domain familiarity as a cue for these judgments. For example, both Koriat’s (1997) cue-utilization theory and Dunlosky and Tauber’s (2014) isomechanism theory rely on the assumption that people have a priori information (conscious or not) about the predictive validity of a cue and incorporate that cue into their judgments accordingly. Such theories can account for the basic linear relationship between participants’ domain familiarity and their JOLs. The isomechanism theory (and to a less explicit extent, the cue-utilization theory) provides two major routes by which a cue can affect JOLs. First, participants might judge facts from better-known topics to be better-learned than facts from lesser-known topics because of a nonconscious experience that differs by domain familiarity. For example, while studying facts from better-known topics, people might experience greater processing fluency or more related information might be activated from long-term memory compared to when studying facts from lesser-known topics (cf. Koriat and Ackerman 2010; Koriat et al. 2008; Serra and Magreehan 2016; Serra and McNeely 2020). People might (consciously or not) associate these experiences with greater memory and make JOLs accordingly. Second, participants might judge facts from better-known topics to be better-learned than facts from lesser-known topics because they hold the explicit belief that domain familiarly relates to memory and incorporate it into their JOLs in a conscious and purposeful way (cf. Briñol et al. 2006; Frank and Kuhlmann 2017; Hu et al. 2015; Mueller et al. 2013, 2014; Serra and Ariel 2014; Serra and Dunlosky 2010; Su et al. 2018). Either path could explain the linear relationship between JOLs and domain familiarity, and either could also lead to the overutilization of this cue for JOLs. Critically, however, neither theory easily predicts that the overutilization pattern (or even the simple linear relationship between JOLs and domain familiarity) would be affected by aspects of the procedure such as whether participants study the facts blocked by topic or not.

In contrast, the analytic processing theory proposed by Mueller and Dunlosky (2017) assumes that participants sometimes enter a task without clear expectations about how a cue might relate to memory. In such situations, participants might engage in analytic thinking that leads them to seek out cues to help make their JOLs, regardless of the predictive validity of those cues. For example, Mueller and Dunlosky (2017) led participants to believe that the color of the font that memory materials appeared in would affect their memory for the items. With little other information to use for their JOLs, participants began to use font color as a basis for their JOLs even though font color did not actually affect their memory for the items. Such cue utilization is likely emergent and therefore might sometimes be affected by the conditions of the task. Consider as well that font size does not usually affect memory, yet participants often judge larger-font items to be more memorable than smaller-font items. Magreehan et al. (2016) found that participants would only use font size as a cue for their JOLs when they experienced more than one level of font size, indicating that the effect of font size on JOLs likely reflected some form of analytical thinking (e.g., “I think the larger items are more memorable than the smaller items”) rather than a difference in experience that could occur with only one level of font size (e.g., greater visual processing fluency when studying larger than smaller items).

Given the assumptions of analytic processing theory, in the present studies we sought to identify factors that might lead people to exhibit the overutilization pattern when studying facts across multiple topics. We hypothesized that some conditions of the task—such as whether participants studied facts blocked by topic or not—might make them more or less likely to demonstrate this pattern.

#### 1.1.3. Relative Accuracy as a Function of Domain Familiarity

Participants’ learning and JOLs are often positively related to their domain familiarity. Therefore, using domain familiarity as a cue for JOLs should be a useful and predictive cue for learners to consider when making JOLs across topics. That said, some researchers have also been interested in whether greater domain knowledge allows learners to demonstrate greater relative accuracy within topics (e.g., Glenberg and Epstein 1987; Witherby et al. 2023). One major assumption is that having greater expertise about a topic would make learners better able to discriminate their learning within that topic than within a topic they know less about (cf. Glenberg and Epstein 1987; see also Kruger and Dunning 1999 for similar logic). Surprisingly, few studies have tested this relationship. Many of those that have tested this possibility have examined reading comprehension (rather than memory-focused measures of learning), and often only for two topics. To further complicate the situation, the findings to date have been inconsistent. Whereas some studies have found no relationship between domain familiarity and within-topic relative accuracy (i.e., Griffin et al. 2009; Shanks and Serra 2014), others have found a negative association between domain knowledge and relative accuracy (e.g., Glenberg and Epstein 1987; Witherby et al. 2023). For example, Glenberg and Epstein (1987) compared comprehension and judgments for physics majors and music majors studying information from only those two domains. Overall, participants’ within-topic relative accuracy was negatively correlated with their topic expertise. More recently, in a study that was conceptually similar to that of Glenberg and Epstein (1987), Witherby et al. (2023) had people with varied levels of knowledge about cooking and American football learn novel (i.e., made-up) facts from one of the two domains for a later test. Much as in Glenberg and Epstein’s study, participants’ prior knowledge was negatively related to the relative accuracy of their judgments. In contrast, Griffin et al. (2009) compared comprehension and judgments for people with high and low baseball knowledge after reading texts about baseball and texts on other topics. In this case, the researchers found no difference in relative accuracy by topic expertise. Shanks and Serra (2014) also did not find a relationship between domain familiarity and within-topic relative accuracy, but their participants studied 100 different facts from 10 different topics for a memory test. Although there is no obvious reason to expect topic expertise to function differently in relation to metamemory or metacomprehension processes, without other similar memory studies to compare to, the Shanks and Serra study is currently the major data point to this end.

As we noted above, calculating a gamma correlation across items between a participants’ JOLs and recall outcomes is a common way to assess relative accuracy. Gamma, however, is sometimes incalculable if either measure shows low variance. Importantly, consider a situation like Shanks and Serra’s (2014) methodology where each participant studied and judged facts from 10 different domains. Researchers can attempt to calculate a relative accuracy score for each of the 10 topics for each participant, but a missing value for any single topic would eliminate that participant from the entire analysis. As noted by Witherby et al. (2023), only 10 of 50 participants in Shanks and Serra’s (2014) first experiment had calculable relative accuracy scores for all 10 topics. When examining scores only for those 10 participants, relative accuracy did not differ by topic ranking, *F*(9,81) = 0.917, *MSE* = 0.233, *p* = .515, *η_p_*^2^ = .092. Concordantly, there was no linear relationship between relative accuracy and domain familiarity (*p* = .333, *η_p_*^2^ = .104).

The main reason that a gamma correlation might not be calculable is that one (or both) of the variables being correlated show little or no variance. As an example, consider participants who correctly recall all items; even if their JOLs are quite varied, there is no variance for the recall measure, so a gamma correlation cannot be calculated. The participants could not have assigned higher JOLs to recalled items and lower JOLs to nonrecalled items, because they recalled all the items. In Shanks and Serra’s (2014) studies and in the present studies, a gamma correlation could be calculated between JOLs and recall for each topic (i.e., 10 values per participant). Analyzing these scores with a repeated-measures ANOVA requires a participant to have a score for each topic. If a participant is missing even one score, he or she would be completely excluded from the analysis. As such, an alternative is to input values of zero for any gamma correlation that could not be calculated due to low variance. The value of zero conveys the same information as a missing value—there is no ordinal relationship between the two variables—but allows all participants to be included in the analysis.

To examine the relationship between relative accuracy and domain familiarity further, we input values of zero for any gamma correlation that could not be calculated. All means were still significantly greater than zero (all *p*s < .001), indicating that participants’ relative accuracy within topics was better than chance after adding in the missing values and that inputting values of zero did not artificially deflate these scores beyond consideration (see Griffin et al. 2009, for some discussion of the need to have means above zero). These new means appear in Figure 1c. Although relative accuracy still did not differ by topic ranking, *F*(9,441) = 1.014, *MSE* = 0.226, *p* = .428, *η_p_*^2^ = .020, the linear relationship was significant, (*p* = .008, *η_p_*^2^ = .137), with relative accuracy higher for better-known topics than for lesser-known topics. As inputting 0 s for missing values allowed us to consider relative accuracy more fully under the current paradigm, we also used this strategy in the subsequent studies.

We can consider this reanalysis tentatively, but it suggests that in some cases, relative accuracy might be related to people’s domain familiarity, such that they can better discriminate their learning within topics they know more about than within topics they know less about (cf. Glenberg and Epstein 1987). Given that few studies have compared the relative accuracy of people’s learning judgments across two or more domains and results to date have been inconsistent, we examined the potential relationship between domain familiarity and relative accuracy in the present studies. None of the theories of metacognitive monitoring we reviewed above (i.e., Dunlosky and Tauber 2014; Koriat 1997; Mueller and Dunlosky 2017) makes any explicit predictions about how domain knowledge might relate to metacognitive accuracy. But, as these theories all rely on cue utilization to explain metacognitive accuracy, it might be assumed that people who know more about a topic also have more advanced knowledge of cue validity within that topic than do people who know less about a topic. Such a difference could produce a positive relationship between domain familiarity and relative accuracy (cf. Glenberg and Epstein 1987; Kruger and Dunning 1999).

### 1.2. The Present Studies

We conducted the present studies in accordance with the Declaration of Helsinki. They were approved by the Institutional Review Board of Texas Tech University (“The Effect of Expertise on Judgments of Learning”) on 7 February 2010. We have uploaded the materials and data for the present studies to https://osf.io/fn2z8/.

Specifically, we examined the relationship between participants’ domain familiarity, their learning of facts from 10 different domains, and the accuracy of their JOLs for those facts. We based the basic paradigm on Shanks and Serra’s (2014) methodology: participants rank ordered their preexisting knowledge across 10 different domains, studied and judged facts from those domains, and then completed a test over the facts. In Study 1, we examined the potential influence of participants’ metacognitive beliefs on the overutilization pattern by warning half the participants to avoid this bias in their judgments. In Study 2, we examined the potential influence of the rank-order procedure on the overutilization pattern by having half the participants wait until the end of the task to rank order their expertise in the topics. In Studies 3 and 4, we examined the potential influence of participants studying the facts blocked by topic by having half the participants in Study 3 study the facts without blocking by topic and by having the participants in Study 4 only study facts from one topic. In all studies, we considered whether the relative accuracy of participants’ JOLs was related to their domain familiarity across topics, and whether any of the factors noted above altered the relationship between relative accuracy and domain familiarity. We discuss the specific rationale of each study in more detail in turn.

## 2. Study 1

As we reviewed above, participants often factor their beliefs about cognition—correctly or not—into their JOLs (Dunlosky and Tauber 2014; Mueller and Dunlosky 2017). In the present situation, people might overutilize domain familiarity as a cue for JOLs because they explicitly apply beliefs about their domain knowledge to their judgments. A student might believe that they know more about Biology than they do about Math, and that they, therefore, might be better able to learn new facts about Biology than about Math. Although learning is indeed often better for facts from topics people already know more about (Witherby and Carpenter 2022), they might apply this belief too strongly, resulting in judgments that greatly overpredict their learning of new Biology facts (cf. Glenberg et al. 1987).

Before we could examine the (presumably nonconscious) effects of task experience on the overutilization pattern, we first wanted to consider if the pattern reflected participants’ application of beliefs about domain familiarity to their JOLs. To do so, in Study 1 we explained the overutilization pattern to half the participants and warned them not to utilize domain familiarity as a basis for their JOLs (cf. Koriat and Bjork 2006). If participants explicitly apply beliefs about their domain knowledge to make JOLs, then warning them not to do so could reduce or eliminate the overutilization pattern—and even the overall association between ranking and JOLs—compared to unwarned participants.

### 2.1. Method

#### 2.1.1. Participants

The participants were 100 undergraduate students from Texas Tech University. They participated online for course credit. When we conducted the present studies, we based our sample sizes on those of Shanks and Serra (2014). But, as detecting a potential interaction for our absolute accuracy measure was of primary interest, we should note that power analysis in G*Power (Faul et al. 2007) indicated that 100 participants (50 per group) would allow us to detect an interaction with an effect size as small as *η_p_*^2^ = .017.

#### 2.1.2. Materials

The materials were the same 100 facts and 100 test questions from Shanks and Serra (2014). Ten facts/questions stemmed from each of 10 different topics (atmospheric science, biology, business, chemistry, English, history, math, performing arts, psychology, and sociology). All 100 facts were adapted from undergraduate textbooks on these topics.

#### 2.1.3. Design

Study 1 used a 2 (warning vs. no warning) × 10 (topic ranking: 1 (most known) to 10 (least known)) mixed design, with warning as a between-participants independent variable and topic ranking as a within-participants quasi-independent variable. The dependent variables were recall performance (percent correct), JOL magnitude, absolute accuracy (JOLs minus recall), and relative accuracy (gamma correlations between JOLs and recall).

#### 2.1.4. Procedure

The procedure for the No-Warning Group was the same as the procedure for Experiment 1 in Shanks and Serra (2014). Specifically, the entire procedure occurred online using Qualtrics. After providing informed consent, participants read instructions describing the study–judgment–test procedure they would be completing. They then rank ordered the 10 topics (atmospheric science, biology, business, chemistry, English, history, math, performing arts, psychology, sociology) from their best-known topic (ranked 1) to their least-known topic (ranked 10). The topics appeared onscreen in a random order for each participant to avoid order effects, but all were always present on the screen; participants indicated their rank ordering by moving the topics up or down until their best-known topic was first and their least-known topic was last. Participants then studied the 100 facts (e.g., from biology: “Karyokinesis is the division of the nucleus during the cell cycle”), organized into blocks of 10 facts by topic, in a random order of topics and facts within each topic for each participant. After studying each fact, they made a JOL indicating the likelihood that they would be able to answer a question on that fact on the subsequent test, using a 0% to 100% likelihood scale. They typed their JOL into a field on the computer screen, which would only accept whole number values from 0 to 100. Participants studied each fact one at a time and at their own pace, but they could not go back to restudy any previous facts. After studying all 100 facts, participants completed a test on their learning of the facts, answering one question at a time until they responded to all 100 questions (e.g., “What is the division of the nucleus during the cell cycle called?”). They typed their answer into a field on the computer screen (i.e., “karyokinesis”), or left it blank to skip it. As with the study and judgment phase, the test phase proceeded in blocks by topic, with all 10 questions from a topic occurring together. The order of the blocks and the questions within the blocks was randomized for each participant, independently from the study order. After completing the test, participants read a short debriefing and then received their course credit automatically.

The procedure for the Warning Group was identical to the procedure for the No-Warning Group except that, just before these participants began the study and JOL phase of the task, they read the following warning: “Participants often make their judgments for each item based on their familiarity with the topic area that the fact comes from. Put differently, they tend to make their judgments for each specific piece of information based on how much they already know about the topic area in general rather than how well they have learned that specific piece of information. Try not to do this when you make your judgments for each item! Instead, for each piece of information, try to focus on how well you know that single piece of information regardless of what topic it is a part of. Try not to let your judgments be biased by your existing knowledge of that topic in general.”

### 2.2. Results

Note that for all analyses in all the studies, we used each participants’ rank ordering of the 10 topics from their personally best-known topic (1) to least-known topic (10) as a quasi-independent variable. We did not have any hypotheses about the specific topics, so we did not analyze any dependent variable based on the specific topics. Therefore, the same topic (e.g., sociology) could have been the best-known topic for one participant and the least-known topic for another participant.

#### 2.2.1. Recall Performance

Most correct answers involved either a single word or a short phrase, so there was not much variance in the responses provided by participants. In this and all other studies, we therefore scored answers as correct or incorrect; we did not award any partial credit.

**Figure 2:**
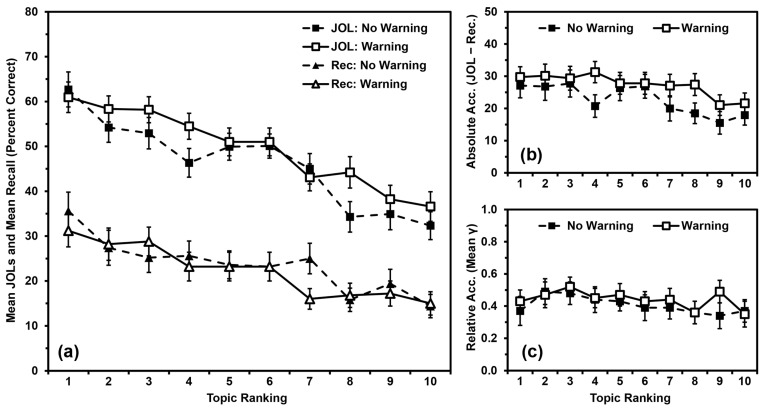
Study 1 results: (**a**) Mean JOLs and mean recall performance (percent correct) by topic ranking; (**b**) Mean absolute accuracy (JOLs minus recall) by topic ranking; (**c**) Mean relative accuracy (gamma correlations) by topic ranking after inputting 0 s for missing values. Error bars are one standard error of the mean.

For each participant, we calculated the percentage of questions they answered correctly for each topic ranking (Figure 2a). We analyzed recall performance using a 2 (warning vs. no warning) × 10 (topic ranking: 1 (most known) to 10 (least known)) mixed ANOVA, with warning as a between-participants independent variable and topic ranking as a within-participants quasi-independent variable. Recall did not differ by warning, *F*(1,98) = 0.140, *MSE* = 2750.669, *p* = .709, *η_p_*^2^ = .001. Recall differed by topic ranking, *F*(9,882) = 12.419, *MSE* = 260.697, *p* < .001, *η_p_*^2^ = .112, with a polynomial contrast indicating that recall performance was linearly related to topic ranking (*p* < .001). Warning and ranking did not interact, *F*(9,882) = 1.180, *MSE* = 260.697, *p* = .305, *η_p_*^2^ = .012.

#### 2.2.2. JOL Magnitude

For each participant, we calculated the mean JOL for each topic ranking (Figure 2a). We analyzed JOLs using a 2 (warning vs. no warning) × 10 (topic ranking: 1 (most known) to 10 (least known)) mixed ANOVA. JOLs did not differ by warning, *F*(1,98) = 0.945, *MSE* = 2944.033, *p* = .333, *η_p_*^2^ = .010. JOLs differed by topic ranking, *F*(9,882) = 33.049, *MSE* = 249.128, *p* < .001, *η_p_*^2^ = .252, with a polynomial contrast indicating that JOLs were linearly related to topic ranking (*p* < .001). Warning and ranking did not interact, *F*(9,882) = 1.512, *MSE* = 249.128, *p* = .139, *η_p_*^2^ = .015.

#### 2.2.3. Absolute Accuracy

For each participant, we calculated the absolute accuracy (mean JOL minus mean recall) for each topic ranking (Figure 2b). We analyzed absolute accuracy using a 2 (warning vs. no warning) × 10 (topic ranking: 1 (most known) to 10 (least known)) mixed ANOVA. Absolute accuracy did not differ by warning, *F*(1,98) = 1.442, *MSE* = 3630.634, *p* = .233, *η_p_*^2^ = .015. Absolute accuracy differed by topic ranking, *F*(9,882) = 4.680, *MSE* = 299.651, *p* < .001, *η_p_*^2^ = .046, with a polynomial contrast indicating that absolute accuracy was linearly related to topic ranking (*p* < .001). Warning and ranking did not interact, *F*(9,882) = 0.920, *MSE* = 299.651, *p* = .507, *η_p_*^2^ = .009. That said, to provide analyses in line with those of Studies 2 and 3, we also examined the relationship between absolute accuracy and topic ranking within each group. In line with the omnibus analysis, absolute accuracy was linearly related to topic ranking for both the No-Warning Group (*p* = .003, *η_p_*^2^ = .162) and the Warning Group (*p* = .004, *η_p_*^2^ = .154).

#### 2.2.4. Relative Accuracy

For each participant, we calculated the relative accuracy (a gamma correlation between JOLs and recall) for each topic ranking (Figure 2c). Gamma is sometimes incalculable if one or both measures show low variance and a missing value for any single topic would eliminate that participant from the entire analysis, so we input values of zero for any gamma correlation that could not be calculated due to low variance. All means were significantly greater than zero (all *p*s < .001), indicating that participants’ relative accuracy within topics was better than chance, and that inputting values of zero did not artificially deflate these scores beyond consideration. We then analyzed relative accuracy using a 2 (warning vs. no warning) × 10 (topic ranking: 1 (most known) to 10 (least known)) mixed ANOVA. Relative accuracy did not differ by warning, *F*(1,98) = 0.532, *MSE* = 0.634, *p* = .467, *η_p_*^2^ = .005, or by topic ranking, *F*(9,882) = 0.906, *MSE* = 0.225, *p* = .519, *η_p_*^2^ = .009. Warning and ranking did not interact, *F*(9,882) = 0.287, *MSE* = 0.225, *p* = .978, *η_p_*^2^ = .003.

### 2.3. Discussion

The basic results of Study 1 replicated those of Shanks and Serra (2014): recall and JOLs were both linearly related to topic rankings, with participants recalling more facts and making higher JOLs for topics they rated as better known and recalling fewer facts and making lower JOLs for topics they rated as less known. As in that prior research, participants were consistently overconfident, and overconfidence was linearly related to topic rankings. Participants were more overconfident for topics rated as better known and less overconfident for topics rated as less known (i.e., the overutilization pattern). In contrast, relative accuracy was not related to topic rankings, even after inputting 0 s for any missing values.

The warning to avoid overutilizing domain familiarity as a cue for JOLs (cf. Rhodes and Castel 2008; Serra and Metcalfe 2009) did not alter any of these outcomes. Therefore, the tendency for participants to overutilize domain familiarity as a cue for JOLs—and the general relationship of domain familiarity with JOLs and recall—might not reflect the purposeful application of explicit beliefs about domain familiarity to JOLs. In subsequent studies, we therefore tested the idea that the overutilization pattern stems from how participants experience domain familiarity during the task.

## 3. Study 2

In Study 1 and in both of Shanks and Serra’s (2014) studies, the rank-ordering phase of the task preceded the study and JOL phase. Having participants rank order the topics first could have inflated the relationship between rankings and JOLs, leading to the overutilization pattern. To examine this possibility, in Study 2, we varied whether participants completed the rank-ordering phase before or after the task. If completing the ranking first produces the overutilization pattern, then this pattern should be reduced (or even eliminated) for participants who complete the ranking after study and judgment.

### 3.1. Method and Materials

The participants were 100 undergraduate students from Texas Tech University. They participated online for course credit. None had participated in Study 1. As detecting a potential interaction for our absolute accuracy measure was of primary interest, power analysis in G*Power (Faul et al. 2007) indicated that 100 participants (50 per group) would allow us to detect an interaction with an effect size as small as *η_p_*^2^ = .017.

The materials and procedure for the Ranking-First Group in Study 2 were the same as for the No-Warning Group in Study 1. For the Ranking-Last Group, the procedure was the same as for the Ranking-First Group, except that the participants completed rank ordering after studying, making JOLs, and testing of the facts.

### 3.2. Results

#### 3.2.1. Recall Performance

**Figure 3:**
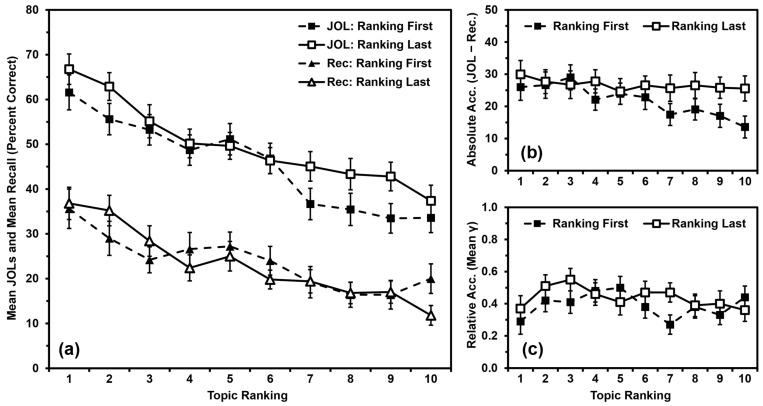
Study 2 results: (**a**) Mean JOLs and mean recall performance (percent correct) by topic ranking; (**b**) Mean absolute accuracy (JOLs minus recall) by topic ranking; (**c**) Mean relative accuracy (gamma correlations) by topic ranking after inputting 0 s for missing values. Error bars are one standard error of the mean.

We analyzed recall performance (Figure 3a) using a 2 (order: ranking first vs. ranking last) × 10 (topic ranking: 1 to 10) mixed ANOVA, with order as a between-participants independent variable and topic ranking as a within-participants quasi-independent variable. Recall did not differ by order, *F*(1,98) = 0.035, *MSE* = 2551.188, *p* = .851, *η_p_*^2^ < .001. Recall differed by topic ranking, *F*(9,882) = 17.264, *MSE* = 272.009, *p* < .001, *η_p_*^2^ = .150, with a polynomial contrast indicating that recall performance was linearly related to topic ranking (*p* < .001). Order and ranking did not interact, *F*(9,882) = 1.653, *MSE* = 272.009, *p* = .096, *η_p_*^2^ = .017.

#### 3.2.2. JOL Magnitude

We analyzed JOLs (Figure 3a) using a 2 (order: ranking first vs. ranking last) × 10 (topic ranking: 1 to 10) mixed ANOVA, with order as a between-participants independent variable and topic ranking as a within-participants quasi-independent variable. JOLs did not differ by order, *F*(1,98) = 1.293, *MSE* = 3617.258, *p* = .258, *η_p_*^2^ = .013. JOLs differed by topic ranking, *F*(9,882) = 38.121, *MSE* = 237.537, *p* < .001, *η_p_*^2^ = .280, with a polynomial contrast indicating that JOLs were linearly related to topic ranking (*p* < .001). Order and ranking did not interact, *F*(9,882) = 1.578, *MSE* = 237.537, *p* = .117, *η_p_*^2^ = .016.

#### 3.2.3. Absolute Accuracy

We analyzed absolute accuracy (Figure 3b) using a 2 (order: ranking first vs. ranking last) × 10 (topic ranking: 1 to 10) mixed ANOVA, with order as a between-participants independent variable and topic ranking as a within-participants quasi-independent variable. Absolute accuracy did not differ by order, *F*(1,98) = 1.542, *MSE* = 3932.156, *p* = .217, *η_p_*^2^ = .015. Absolute accuracy differed by topic ranking, *F*(9,882) = 2.485, *MSE* = 336.739, *p* = .008, *η_p_*^2^ = .025, with a polynomial contrast indicating that absolute accuracy was linearly related to topic ranking (*p* < .001). Order and ranking did not interact, *F*(9,882) = 1.393, *MSE* = 336.739, *p* = .187, *η_p_*^2^ = .014.

Although the interaction was not significant in the omnibus ANOVA, the obtained effect size of *η_p_*^2^ = .014 was just shy of our calculated effect size for a significant interaction of *η_p_*^2^ = .017. Additionally, Figure 3b suggests that the relationship between absolute accuracy and topic ranking might have differed between groups, and the polynomial contrast indicated that the two groups’ linear relationships to topic ranking differed (*p* = .040). Indeed, absolute accuracy was linearly related to topic ranking for the Ranking-First Group (*p* < .001, *η_p_*^2^ = .313), but not for the Ranking-Last Group (*p* = .424, *η_p_*^2^ = .013). We have provided these analyses for completeness, but we will interpret this interaction as being “not significant” in the remainder of the paper per the standards of null-hypothesis significance testing (NHST).

#### 3.2.4. Relative Accuracy

We input values of zero for any gamma correlation that could not be calculated due to low variance. All means were significantly greater than zero (all *p*s < .001), indicating that participants’ relative accuracy within topics was better than chance, and that inputting values of zero did not artificially deflate these scores beyond consideration. We then analyzed relative accuracy (Figure 3c) using a 2 (order: ranking first vs. ranking last) × 10 (topic ranking: 1 to 10) mixed ANOVA, with order as a between-participants independent variable and topic ranking as a within-participants quasi-independent variable. Relative accuracy did not differ by order, *F*(1,98) = 1.198, *MSE* = 0.525, *p* = .276, *η_p_*^2^ = .012, or by topic ranking, *F*(9,882) = 1.175, *MSE* = 0.224, *p* = .308, *η_p_*^2^ = .012. Order and ranking did not interact, *F*(9,882) = 0.963, *MSE* = 0.224, *p* = .470, *η_p_*^2^ = .010.

### 3.3. Discussion

Participants’ recall and JOLs were linearly related to their rank ordering of the topics regardless of whether they completed the rank-ordering phase before or after the study and JOL phase. Relative accuracy was again not related to rank ordering.

The marginal absolute-accuracy results in Study 2 could suggest that rank ordering the topics before completing the primary task contributes to the overutilization pattern, but we did not have enough power to detect the interaction. We should note, however, that in Studies 3 and 4, we were able to eliminate the overutilization pattern by not having participants study facts blocked by topic, even after completing the topic ranking. Completing the topic ranking first might contribute to the overutilization pattern, but if so, it is not a strong enough effect to produce the overutilization pattern on its own.

## 4. Study 3

In the present Studies 1 and 2 (and in Shanks and Serra 2014), participants studied the facts blocked by topic. Having participants study facts blocked by topic could have inflated the relationship between rankings and JOLs, leading to the overutilization pattern. To examine this possibility, in Study 3, we varied whether participants studied and judged the facts blocked by topic or in a completely random order with no blocking. If blocking produces the overutilization pattern, then this pattern should be reduced (or even eliminated) for participants who study the facts without blocking by topic.

### 4.1. Method and Materials

The participants were 100 undergraduate students from Texas Tech University. They participated online for course credit. None had participated in Studies 1 or 2. As detecting a potential interaction for our absolute accuracy measure was of primary interest, power analysis in G*Power (Faul et al. 2007) indicated that 100 participants (50 per group) would allow us to detect an interaction with an effect size as small as *η_p_*^2^ = .017.

The materials and procedure for the Blocking Group in Study 3 were the same as for the No-Warning Group in Study 1 and the Ratings-First Group in Study 2. For the No-Blocking Group, the procedure was the same as for the Blocking Group except that the participants studied and judged the 100 facts in a random order with no blocking by topic and later tested over the 100 facts in a random order with no blocking by topic.

### 4.2. Results

#### 4.2.1. Recall Performance

**Figure 4:**
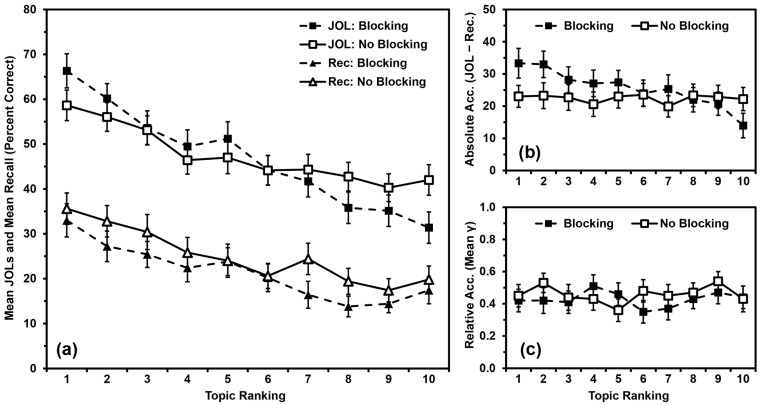
Study 3 results: (**a**) Mean JOLs and mean recall performance (percent correct) by topic ranking; (**b**) Mean absolute accuracy (JOLs minus recall) by topic ranking; (**c**) Mean relative accuracy (gamma correlations) by topic ranking after inputting 0 s for missing values. Error bars are one standard error of the mean.

We analyzed recall performance (Figure 4a) using a 2 (blocking vs. no blocking) × 10 (topic ranking) mixed ANOVA, with blocking as a between-participants independent variable and topic ranking as a within-participants quasi-independent variable. Recall did not differ by blocking, *F*(1,98) = 1.351, *MSE* = 2425.406, *p* = .248, *η_p_*^2^ = .014. Recall differed by topic ranking, *F*(9,882) = 12.840, *MSE* = 282.572, *p* < .001, *η_p_*^2^ = .116, with a polynomial contrast indicating that recall performance was linearly related to topic ranking (*p* < .001). Blocking and ranking did not interact, *F*(9,882) = 0.530, *MSE* = 282.572, *p* = .853, *η_p_*^2^ = .005.

#### 4.2.2. JOL Magnitude

We analyzed JOLs (Figure 4a) using a 2 (blocking vs. no blocking) × 10 (topic ranking: 1 to 10) mixed ANOVA, with blocking as a between-participants independent variable and topic ranking as a within-participants quasi-independent variable. JOLs did not differ by blocking, *F*(1,98) = 0.019, *MSE* = 4158.770, *p* = .889, *η_p_*^2^ < .001. JOLs differed by topic ranking, *F*(9,882) = 40.650, *MSE* = 187.063, *p* < .001, *η_p_*^2^ = .293, with a polynomial contrast indicating that JOLs were linearly related to topic ranking (*p* < .001). Blocking and ranking interacted significantly, *F*(9,882) = 4.378, *MSE* = 187.063, *p* < .001, *η_p_*^2^ = .043. Additionally, the linear relationship between JOLs and topic ranking varied by group (*p* < .001). Specifically, JOLs were linearly related to topic ranking in both groups, but the effect size was slightly larger for the Blocking Group (*p* < .001, *η_p_*^2^ = .624) than for the No-Blocking Group (*p* < .001, *η_p_*^2^ = .604).

#### 4.2.3. Absolute Accuracy

We analyzed absolute accuracy (Figure 4b) using a 2 (blocking vs. no blocking) × 10 (topic ranking: 1 to 10) mixed ANOVA, with blocking as a between-participants independent variable and topic ranking as a within-participants quasi-independent variable. Absolute accuracy did not differ by blocking, *F*(1,98) = 0.479, *MSE* = 4863.133, *p* = .491, *η_p_*^2^ = .005. Absolute accuracy differed by topic ranking, *F*(9,882) = 3.236, *MSE* = 279.248, *p* < .001, *η_p_*^2^ = .032, with a polynomial contrast indicating that absolute accuracy was linearly related to topic ranking (*p* < .001). Blocking and ranking interacted significantly, *F*(9,882) = 3.007, *MSE* = 279.248, *p* = .002, *η_p_*^2^ = .030. Additionally, the linear relationship between absolute accuracy and topic ranking also varied by group (*p* < .001). Specifically, absolute accuracy was linearly related to topic ranking for the Blocking Group (*p* < .001, *η_p_*^2^ = .299), but not for the No-Blocking Group (*p* = .836, *η_p_*^2^ = .001).

#### 4.2.4. Relative Accuracy

We input values of zero for any gamma correlation that could not be calculated due to low variance. All means were significantly greater than zero (all *p*s < .001), indicating that participants’ relative accuracy within topics was better than chance, and that inputting values of zero did not artificially deflate these scores beyond consideration. We then analyzed relative accuracy (Figure 4c) using a 2 (blocking vs. no blocking) × 10 (topic ranking: 1 to 10) mixed ANOVA, with blocking as a between-participants independent variable and topic ranking as a within-participants quasi-independent variable. Relative accuracy did not differ by blocking, *F*(1,98) = 0.391, *MSE* = 0.571, *p* = .533, *η_p_*^2^ = .004, or by topic ranking, *F*(9,882) = 0.468, *MSE* = 0.214, *p* = .896, *η_p_*^2^ = .005. Blocking and ranking did not interact, *F*(9,882) = 0.691, *MSE* = 0.214, *p* = .717, *η_p_*^2^ = .007.

### 4.3. Discussion

Participants’ recall and JOLs were linearly related to their rank ordering of the topics regardless of whether they studied the facts blocked by topic or in a random order. Relative accuracy was again not related to rank ordering. Absolute accuracy, however, was only linearly related to topic ranking for the Blocking Group; the No-Blocking Group did not show a pattern indicating overutilization of domain familiarity as a cue for JOLs (Figure 4b). This outcome suggests that something about the experience of studying the facts blocked by topic contributes to the overutilization pattern.

According to analytic processing theory (Mueller and Dunlosky 2017), making metacognitive judgments prompts analytic thinking and a search for cues to use to judge their cognition. In the present case, studying the facts blocked by topic might make domain familiarity more salient to participants, and they then incorporate it into their JOLs to a greater extent than when the facts are not blocked by topic. Although this conclusion makes sense considering the results of Study 3, it is difficult to resolve this conclusion with that of Study 1, which suggests that participants do not purposely employ an explicit belief about domain familiarity to produce the pattern. Instead, the experience of studying the facts blocked by topic might generate an experiential cue that leads participants to overutilize domain familiarity as cue for JOLs but that is not present when they study facts in a random order. Research on category learning suggests that people experience greater ease or fluency when they study exemplars from the same category in succession as opposed to intermixed (e.g., Kornell and Bjork 2008a; Yan et al. 2016). Studying facts from the same topic in a block might produce a similar sense of fluency as does studying exemplars from the same category. This idea can help explain why warning participants not to overutilize domain familiarity as a cue (Study 1) and moving the rank-ordering phase (Study 2) did not eliminate the overutilization pattern; studying the facts in blocks could still produce a subjective experience that led to the pattern. We examined this possibility further in Study 4.

## 5. Study 4

The results of Study 3 suggest that studying facts blocked by topic is a major contributor to the overutilization of domain familiarity as a cue for JOLs. In Study 4, we tested this idea further by having participants only study facts from one topic (either the topic they ranked as their first, fourth, seventh, or tenth best-known topic). If studying facts blocked by topic is a major contributor to the overutilization pattern, then only studying facts from one topic should reduce or eliminate it as happened in Study 3 when participants studied all the facts intermixed.

Study 4 also allowed for a better test of the relationship between relative accuracy and domain familiarity, as participants only studied facts from one topic, and they studied 20 facts instead of 10 as in the other studies. These conditions should increase the chance that a linear relationship between domain familiarity and relative accuracy could occur.

### 5.1. Method and Materials

The participants were 160 undergraduate students from Texas Tech University. They participated online for course credit. None had participated in Studies 1, 2, or 3. As detecting a potential interaction for our absolute accuracy measure was of primary interest, power analysis in G*Power (Faul et al. 2007) indicated that 160 participants (40 per group) would allow us to detect an interaction with an effect size as small as *η_p_*^2^ = .010.

Study 4 was very similar to the prior studies, but also involved significant changes. We created another 100 facts (10 per topic), so each topic had 20 facts. As in the prior studies, participants first rank ordered the 10 topics from their best-known topic to their least-known topic. The survey software then randomly assigned each participant to study 20 facts from only one topic: either the topic they rated as their first best-known topic, fourth best-known topic, seventh best-known topic, or tenth best-known topic (i.e., their least-known topic). They studied and made JOLs for those 20 facts in a random order, then tested on just those 20 facts in a new random order.

### 5.2. Results

#### 5.2.1. Recall Performance

**Figure 5:**
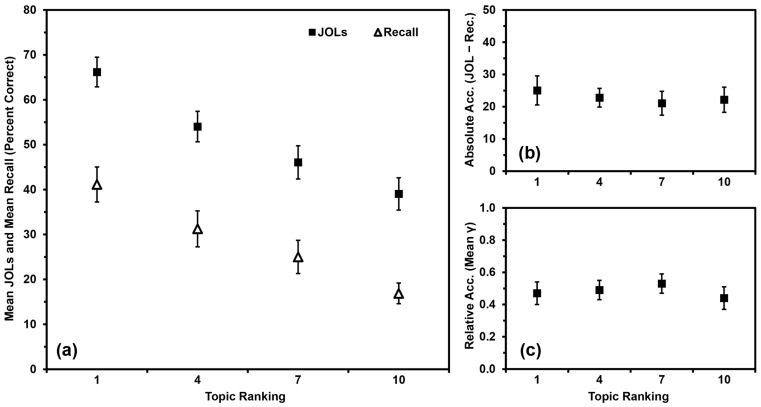
Study 4 results: (**a**) Mean JOLs and mean recall performance (percent correct) by topic ranking; (**b**) Mean absolute accuracy (JOLs minus recall) by topic ranking; (**c**) Mean relative accuracy (gamma correlations) by topic ranking after inputting 0 s for missing values. Error bars are one standard error of the mean.

We analyzed recall performance (Figure 5a) using a between-participants ANOVA with ranking (1, 4, 7, 10) as the only quasi-independent variable. Recall differed by ranking, *F*(3,156) = 8.271, *MSE* = 506.707, *p* < .001, *η_p_*^2^ = .137, with a polynomial contrast indicating that recall was linearly related to topic ranking (*p* < .001).

#### 5.2.2. JOL Magnitude

We analyzed JOLs (Figure 5a) using a between-participants ANOVA with ranking (1, 4, 7, 10) as the only quasi-independent variable. JOLs differed by ranking, *F*(3,156) = 10.993, *MSE* = 493.461, *p* < .001, *η_p_*^2^ = .175, with a polynomial contrast indicating that JOLs were linearly related to topic ranking (*p* < .001).

#### 5.2.3. Absolute Accuracy

We analyzed absolute accuracy (Figure 5b) using a between-participants ANOVA with ranking (1, 4, 7, 10) as the only quasi-independent variable. Absolute accuracy did not differ by topic ranking, *F*(3,156) = 0.198, *MSE* = 572.690, *p* = .897, *η_p_*^2^ = .004.

#### 5.2.4. Relative Accuracy

We input values of zero for any gamma correlation that could not be calculated due to low variance. All means were significantly greater than zero (all *p*s < .001), indicating that participants’ relative accuracy within topics was better than chance, and that inputting values of zero did not artificially deflate these scores beyond consideration. We then analyzed relative accuracy (Figure 5c) using a between-participants ANOVA with ranking (1, 4, 7, 10) as the only quasi-independent variable. Relative accuracy did not differ by topic ranking, *F*(3,156) = 0.365, *MSE* = 0.168, *p* = .779, *η_p_*^2^ = .007.

### 5.3. Discussion

As predicted by the results of Study 3, having participants in Study 4 only study facts from one topic eliminated the overutilization pattern. Arguably however, participants in Study 4 still studied their facts in a “block”, as all the facts were from the same topic. Only studying facts from one topic, however, might not have produced the same sense of fluency that we suggest studying multiple blocks of facts might produce (cf. Kornell and Bjork 2008a; Yan et al. 2016). As with some other cues, participants might need to experience more than one level of fluency for the pattern to occur (cf. Magreehan et al. 2016; Susser et al. 2013).

In line with the prior studies, we did not find a linear between relative accuracy and domain familiarity. This relationship failed to occur even though the design of Study 4 (participants studying 20 facts from only one topic) might have been the most likely to allow this relationship to be found (across groups).

## 6. General Discussion

### 6.1. Findings and Conclusions

In the present studies, we further explored the relationship between domain familiarity and the accuracy of JOLs. We examined whether factors such as participants’ metacognitive beliefs (Study 1), performing the rank-order phase before the study and judgment phase (Study 2), and studying facts blocked by topic (Studies 3 and 4) contribute to the occurrence of the “overutilization of domain familiarity as a cue for JOLs” pattern. We also examined whether a linear relationship between participants’ domain familiarity and the relative accuracy of their JOLs would occur consistently (if at all).

#### 6.1.1. Domain Familiarity as a Cue for JOLs

Our primary research question involved examining factor(s) that might contribute to the occurrence of the overutilization pattern. The results of the present studies closely replicated the findings from Shanks and Serra (2014). Three of the present groups (the No-Warning Group in Study 1, the Ranking-First Group in Study 2, and the Blocking Group in Study 3) were all methodologically identical to each other and to Shanks and Serra’s (2014) Experiment 1. These groups all demonstrated positive relationships between participants’ domain familiarity and their recall, JOLs, and absolute accuracy (i.e., demonstrating the overutilization pattern). So, it seems that the patterns in Figure 1a,b replicate well, at least when the procedure is not altered.

In Study 1, we instructed one group of participants—the Warning Group—to avoid overutilizing domain familiarity as a cue for their JOLs. Nevertheless, they showed the same patterns as the No Warning Group, including a linear relationship between their domain familiarity and absolute accuracy, which suggests that they overutilized this cue for JOLs despite the instructions to avoid doing so. This finding sheds some doubt on the possibility that participants directly apply explicit metacognitive beliefs about the relationship between domain familiarity and learning to make JOLs, although certainly this possibility could be explored using other methods to assess metacognitive beliefs.

The negative result in Study 1 led us to focus on aspects of the procedure that might make the overutilization pattern more or less likely to occur. In Study 2, we varied whether participants rank ordered the topics to indicate their domain familiarity before study and judgment (Ranking-First Group) or after the test (Ranking-Last Group), but the occurrence of the overutilization pattern did not differ. In Study 3, we varied whether participants studied the facts organized into blocks by topic (Blocking Group) or in a random order (No-Blocking Group); the No-Blocking Group did not demonstrate the overutilization pattern. In Study 4, we only had participants study facts from one topic after they indicated their domain familiarity (either their first, fourth, seventh, or tenth best-known topic). Across the four groups, JOLs did not demonstrate the overutilization pattern.

Together, the present results suggest that studying the facts blocked by topic contributes to the occurrence of the overutilization pattern. Of course, there might also be other factors that contribute to the occurrence of the pattern (including, perhaps, some form of metacognitive beliefs), but identifying those other factors and how they all work together to produce this pattern will require additional research. Future research could examine how blocking by topics leads to the overutilization pattern. One possibility is that studying the facts blocked by topic produces greater fluency than does studying the facts in a random order (cf. Kornell and Bjork 2008a; Yan et al. 2016), and this experience affects people’s JOLs in a nonconscious way (Dunlosky and Tauber 2014), leading to the overutilization pattern.

Another possibility, which we did not examine in the present studies, is that studying facts blocked by topic changes how participants convert their confidence in their learning to their JOL values (cf. Higham et al. 2016). For example, England et al. (2017) found that asking participants to make JOLs either in terms of the likelihood of remembering or the likelihood of forgetting does not alter how confident they are in their level of learning; instead, it changes how participants convert (or “scale”) that confidence into an explicit, numerical JOL. In that case, the framing of the judgment produces different patterns of judgment accuracy not because framing affects confidence in memory, but because it affects how people make the judgments. In the present situation, all participants might be using information about the relationship of domain familiarity and recall as a major basis of JOLs, resulting in the positive linear relationships between self-reported domain knowledge, recall, and JOLs. Changes to the procedure, however, might alter how participants scale this relationship to explicit JOL values, producing the overutilization pattern under some conditions but not others. Future research might examine this possibility more directly by using different judgment frames (e.g., likelihood of remembering versus likelihood of forgetting) or different response options (e.g., continuous versus binary responses; see also Hanczakowski et al. 2013; Witherby et al. 2023).

Admittedly, the overutilization pattern might be a very rare occurrence, only occurring when participants are studying information across multiple domains and blocked by topic. But the findings above are still useful for understanding how domain familiarity relates to metacognitive monitoring in general. Based on the present results, aspects of the study design likely alter the experience that learners have while studying, leading them to make JOLs differently and producing the overutilization pattern (or not). The results are compatible with aspects of both analytic processing theory (Mueller and Dunlosky 2017) and isomechanism theory (Dunlosky and Tauber 2014). Both of these theories have routes by which the experience of studying facts in blocks could lead to the overutilization pattern, as well as mechanisms by which that pattern could be altered. The cue-utilization theory (Koriat 1997) can also account for the experience of studying facts in blocks leading to the overutilization pattern but struggles to explain how the pattern was altered in Studies 3 and 4.

#### 6.1.2. Domain Familiarity and Relative Accuracy

Our secondary research question involved considering whether a linear relationship between domain familiarity and relative accuracy would occur consistently using the present paradigm. Findings have been inconsistent as to whether having greater domain familiarity leads to better relative accuracy for learning within that domain (e.g., Glenberg and Epstein 1987; Griffin et al. 2009; Shanks and Serra 2014; Witherby et al. 2023). Whereas Shanks and Serra (2014) initially concluded that domain familiarity and relative accuracy were not linearly related using the present paradigm, our reanalysis of their first experiment in which we input values of zero for any missing gamma correlations suggested that a linear relationship might have in fact been present. Using similar methodology and analyses in the four present studies, however, did not reveal such a trend. Not only did such trends not emerge at the study level, they also did not occur at the group level, even though three of the groups in the present studies (the No-Warning Group in Study 1, the Ranking-First Group in Study 2, and the Blocking Group in Study 3) replicated the method of Shanks and Serra (2014). So, the finding of a linear relationship in Figure 1c might have been spurious. Notably, in none of the present studies did we find a negative relationship as in Glenberg and Epstein (1987) and Witherby et al. (2023).

Participants’ overutilization of domain familiarity as a cue for JOLs might have curbed differences in relative accuracy across topics. In Studies 3 and 4, however, changes to the procedure eliminated the overutilization pattern (i.e., for the No-Blocking Group in Study 3 and the between-groups design of Study 4) but did not lead to linear relationships between relative accuracy and domain familiarity—positive *or* negative. Study 4 might have been the fairest test of whether domain familiarity relates to relative accuracy, as participants only studied and judged facts from one topic and they had 20 facts to judge rather than 10 (per topic), which should have allowed for greater variance of relative-accuracy scores. Even in this case, however, the relationship did not occur.

It is possible that domain familiarity relates to the relative accuracy of people’s metacognitive judgments as originally suggested by Glenberg and Epstein (1987). To date, however, examinations of the association between domain familiarity and relative accuracy have either failed to find a relationship between these variables (e.g., the present studies, Griffin et al. 2009; Shanks and Serra 2014) or have found a negative relationship (e.g., Glenberg and Epstein 1987; Witherby et al. 2023). Unless researchers can identify moderating factors that reliably cause or prevent the relationship from occurring, we might conclude that relative accuracy is not related to domain familiarity but that spurious associations can sometimes be found by chance (e.g., Figure 1c).

### 6.2. Alternative Accounts

Our results suggest that the experience of studying facts blocked by topic contributes to the occurrence of the overutilization pattern in the present paradigm. That said, there are other potential explanations for the pattern that we should address.

Consider the *Dunning–Kruger Effect* (cf. Kruger and Dunning 1999), which involves a form of absolute accuracy for performance predictions. This pattern is identified when the highest-performing people are accurate (or evenly slightly underconfident) at predicting their own performance or judging their own abilities, but the lowest-performing people greatly overestimate their own performance or abilities (i.e., they are said to be “unskilled and unaware”). A common criticism of the Dunning–Kruger Effect is that the stereotypical pattern—the lowest-performing participants make the most overconfident performance judgments—might stem from statistical artefacts such as the hard–easy effect (cf. Burson et al. 2006; Lichtenstein et al. 1982), regression to the mean (Krueger and Mueller 2002), or the imperfect correlation of judgments and performance (Gignac and Zajenkowski 2020). These accounts have been repeatedly debunked as exhaustive explanations for the pattern (cf. Dunning 2011; Ehrlinger et al. 2008; Kruger and Dunning 2002), yet they seem to arise anew every few years (e.g., Gignac and Zajenkowski 2020; Mazor and Fleming 2021).

Readers might wonder whether people’s overutilization of domain familiarity as a cue for JOLs could stem from such statistical artefacts. Regression to the mean and the hard–easy effect attempt to explain the Dunning–Kruger Effect as such: people’s performance predictions tend to be moderate, so when participants’ performance is high, predictions of performance are likely to be underconfident, and when participants’ performance is low, predictions of performance are likely to be overconfident (“unskilled and unaware”). In the present situation, however, such accounts struggle to explain why participants are often the most overconfident for the topic they know best; both regression to the mean and the hard–easy effect would predict a pattern in which participants are overconfident for the topics they know the least about and underconfident for the topics they know the most about. Critics might note that performance in our present studies was fairly low, with recall averaging around 25% across topic rankings and across studies, but with JOLs averaging around 50%. Such accounts could help to explain the persistent overconfidence (although that is more of a description than an explanation), but they cannot explain why the greatest overconfidence occurs for the best-known topics; these accounts would predict less overconfidence for better-known topics (higher performance) and more overconfidence for lesser-known topics (lower performance). Furthermore, such accounts cannot explain why the manipulation in Study 3 eliminated the overutilization pattern without affecting the overall magnitude of participants’ recall or JOLs (versus the comparison group). If the overutilization pattern occurs because JOLs are spuriously “too high” or recall is spuriously “too low”, then reducing or eliminating the occurrence of the pattern should have required lowering JOLs or increasing recall (cf. Burson et al. 2006), neither of which occurred in that study.

By definition, and much like the Dunning–Kruger Effect, the overutilization of domain familiarity pattern is one of imperfect correlation (cf. Gignac and Zajenkowski 2020); but why these correlations are imperfect is the question (see Dunning 2011 for a more detailed discussion). With both effects, claiming that the imperfect correlation of two variables is uninteresting or an illusion because they could simply be imperfectly correlated is circular logic, and does not explain it away. If we expect people’s predictions of their performance or judgments of their abilities to be “perfect”, then it is important to understand when judgments are not accurate. Studies 3 and 4 identified a factor—studying the facts blocked by topics—that might contribute to the occurrence of the imperfect correlation, as the imperfect correlation was eliminated when we removed the blocking.

## 7. Conclusions

The present studies suggest that people might overutilize domain familiarity as a cue for JOLs when studying facts from multiple topics blocked by topic. The results help to explain this effect, but they are also informative for understanding how people make metacognitive judgments in general and what factors can augment or moderate the accuracy of those judgments. The results highlight an important aspect of metacognition that is best described by analytic processing theory (Mueller and Dunlosky 2017): people might search in an analytic fashion during a task to find cues they can use as the basis for their judgments. Importantly, the present results suggest that conditions of the task might affect the outcome of such a search, independent of the validity of the cue in question. For example, although studying items across topics or categories in an intermixed fashion can lead to better learning than studying items blocked by topic or category (e.g., Carvalho and Goldstone 2015; Kornell et al. 2010; Wahlheim et al. 2011), that did not occur in the present Study 3 (i.e., the Blocking and No Blocking groups had equal test performance). Nevertheless, something about experiencing the items blocked by topic led participants to utilize domain familiarity as a cue for their JOLs to a greater extent than did participants who did not study the items blocked by topic. Such effects of task experience on metacognitive judgments—independent of any effects on learning—have been understudied and are not reflected in major theories of metacognitive monitoring which assume the bottom-up selection and utilization based on the cues’ ecological validity (e.g., Koriat 1997; Koriat et al. 2009). Newer theories which posit greater top-down selection of cues (i.e., Mueller and Dunlosky 2017; see also Dunlosky and Tauber 2014) can perhaps better account for such effects.

The original research question (Shanks and Serra 2014) stemmed from a real-world analogue to the present paradigm: students who are studying for multiple final exams in different courses or domains around the same time (or even back-to-back exams). We wondered if students might use their domain familiarity as a guide when judging their learning, deciding for how long to study, and when choosing what to restudy. The results of Shanks and Serra (2014) and the present studies highlighted a substantial irony of this situation: students who are studying for multiple exams around the same time or even in the same day (as is common during “Final Exam Weeks” at many universities) might greatly overestimate their learning for the exam(s) on which they are likely to perform the best. This overestimation could lead students to underprepare for exams they could have scored highest on. Depending on their situation, those courses might be in their major of study, and underperforming on a major course might have greater consequences for their academic standing than underpreparing for an exam in an elective course. As we noted earlier, inaccurate monitoring of learning not only impacts students’ performance for a given exam (e.g., Bjork et al. 2013; Dunlosky and Rawson 2012; Kornell and Bjork 2008b; Kornell and Metcalfe 2006; Metcalfe and Finn 2008), but can also compound over time and lead students to question their abilities, change majors, or drop out (cf. Grimes 2002; Stinebrickner and Stinebrickner 2014). The present results help to identify a potential source of inaccuracy in students’ monitoring of their learning and suggest that studying information blocked by topics might contribute to students overestimating their learning.

## Data Availability

We have uploaded the materials and data for the present studies to https://osf.io/fn2z8/.
